# Supplementary material for: TWIST1 expression is associated with high-risk neuroblastoma and promotes primary and metastatic tumor growth
Source: Commun Biol. 2022 Jan 12;5:42. doi: 10.1038/s42003-021-02958-6 (PMC8755726; doi:10.1038/s42003-021-02958-6)
Supplement: Supplementary file 1 — Supplementary Information [file 42003_2021_2958_MOESM1_ESM.pdf]

Supplementary Information

TWIST1 expression is associated with high-risk neuroblastoma and promotes primary and metastatic tumor growth

Maria-Vittoria Sepporta[1], Viviane Praz[1,2], Katia Balmas Bourloud[1], Jean-Marc Joseph[3], Nicolas Jauquier[3], Nicolo' Riggi[2], Katya Nardou-Auderset[1,4], Audrey Petit[5,6], Jean-Yves Scoazec[7], Hervé Sartelet[5,8], Raffaele Renella[1], Annick Mühlethaler-Mottet[1*]

[1]Pediatric Hematology-Oncology Research Laboratory, Woman-Mother-Child Department, Lausanne University Hospital and University of Lausanne, Switzerland;
[2]Experimental Pathology, Lausanne University Hospital and University of Lausanne, Switzerland;
[3]Pediatric Surgery, Woman-Mother-Child Department, Lausanne University Hospital and University of Lausanne, Switzerland;
[4]Ophthalmic Hospital Jules-Gonin - Fondation Asile Des Aveugles, Lausanne, Switzerland
[5]Department of Pathology, Medical University of Grenoble, Grenoble, France;
[6]Pediatric Hematology Oncology Department, CHU de la Timone, Marseille, France;
[7]Department of Biology and Medical Pathology, Gustave Roussy Institute, Villejuif, France;
[8]Department of Biopathology, CHRU de Nancy, Université de Lorraine, Nancy, France.

*Contact information: Annick.Muhlethaler@chuv.ch

This section includes the description of Supplementary Methods; Supplementary Tables, Supplementary Figures and their Legends; and Supplementary References.

Supplementary Methods:

Cancer Cell Line Encyclopaedia (CCLE) database analysis

The CCLE database (https://portals.broadinstitute.org/ccle) was used to compare the mRNA expression level of TWIST1 and TWIST2 in a range of 36 different cancer types (Affymetrix arrays: Affimetrix Human Genome U133, Plus 2.0). Quality filtering and normalization were performed using Robust Multi-array Average (RMA) and quantile normalization.

TWIST1 knock out through CRISPR/Cas9 technology

Two sgRNAs targeting the first exon of the TWIST1 gene were chosen in the published sgRNA library[1]. Oligos were designed as follow: sgTWIST1-1: forward 5'-CACCGCGGGAGTCCGCAGTCTTACG-3'; reverse 5'-AAACCGTAAGACTGCGGACTCCCGC-3'; sgTWIST1-2: forward 5'-CACCGCTGTCGTCGGCCGGCGAGAC-3'; reverse 5'-AAACGTCTCGCCGGCCGACGACAGC-3'. The lentiviral vector lentiCRISPR v2[2] was a gift from Feng Zhang (Addgene plasmid # 52961; http://n2t.net/addgene:52961; RRID:Addgene_52961) . LentiCRISPR v2-sgTWIST1 plasmids were constructed according to the manufacturer's instructions (Addgene), and used to transduce Control cells. Virus production and lentiviral infections were performed by tranfecting the lentiviral vectors LentiCRISPR v2 or LentiCRISPR v2-sgTWIST1#1 or LentiCRISPR v2-sgTWIST1#2 (10 µg) with 2.5 µg of pMD2.G (a gift from Didier Trono; Addgene plasmid # 12259; http://n2t.net/addgene:12259; RRID:Addgene_12259), and 7.5 µg of psPAX2 (a gift from Didier Trono;Addgene plasmid # 12260; http://n2t.net/addgene:12260; RRID:Addgene_12260) into 293T cells (ATCC GRL-11268) by the CaCl2 method. Forthy-eight hours post-transfection, the lentivirus-containing supernatant were collected, centrifuged at 2.500 rpm at 4°C for 5 min, supplemented with 8 µg/ml of polybrene and filtered through a 0.45 µm filter unit. The filtered solution was used directly to infect the NB cell lines. Transduced SK-N-Be2c, LAN-1 and NB1-M cells were selected 24 h post-infection with either 5 µg/ml for SK-N-Be2c or 1 µg/ml for LAN-1 and NB1-M cells of puromycin (Gibco). Clones were isolated by limiting dilution cloning in a 96-wells plate from the Control and sgTWIST1 #1 of LAN-1 and SK-N-Be2c cell lines. Only clones derived from a single colony were selected and further screened by Immunoblotting. To avoid potential problems caused by variability during single-cell clonal expansion, we pooled 5 and 7 clones for the Control and sgTWIST1 SK-N-Be2c cells, respectively; for the LAN-1 cells, 6 clones were pooled for both Control and sgTWIST1 cells. To note both cell lines are deficient for p53[3] which explain their ability to survive spontaneous apoptosis mediated by MYCN as described in absence of TWIST1[4]. SK-N-Be2c-Control and sgTWIST1 pools of cells were further transduced with a lentiviral vector expressing the mCherry gene (Ex-NEG-Lv244 from GeneCopoeia™). These cells expressing the mCherry

gene were used for the ortho_2 and the sc xenograft experiments. In addition, genome editing in SK-N-Be2c sgTWIST1 cells was verified by NGS sequencing. Briefly, PCR amplicons were designed across the *TWIST1* genomic regions targeted by the sgRNAs to examine generation of indels. A first PCR of 20 cycles was performed using the primers Twist1-nest-F: 5'-GCAAGAAGTCTGCGGGCTGTGG-3' and Twist1-nest-R: 5'-GGATGATCTTCCGCAGCGCG-3', followed by purification with QIAquick PCR purification kit (QIAGEN). Then we run a second PCR of 10 cycles with nested primers containing Illumina overhang adapter sequences: Illumina-P5-Twist1-F: 5'-TCGTCGGCAGCGTCAGATGTGTATAAGAGACAG AAGAAGTCTGCGGGCTGTGGCG-3'; Illumina P7-Twist1-R: 5'-GTCTCGTGGGCTCGGAGATGTGTATAAGAGACAGCGCTCCCGCACGTTGGCCATG-3'. Both PCR were performed using the 2xKAPA HiFi HotStart ReadyMix as follow: 95°C for 5 minutes, 10 cycles at 98°C for 20 s, 73°C for 30 s, 72°C for 30 s; then 72°C for 5 min. Finally, a third PCR was performed to attach Illumina adaptors and barcodes to samples according to manufacturer's instructions. Amplicons were purified by using AMPure XP (Beckman Coulter, Indianapolis, USA), and sequenced with a MiSeq Micro 300 (Illumina Inc., San Diego, USA) at the Lausanne Genomics Technologies Facility (GTF) (https://wp.unil.ch/gtf). After high throughput sequencing reads, PCR amplicons were checked for indels using CRISPResso (https://crispresso.pinellolab.partners.org). The results of the sequencing of the bulk population of SK-N-Be2c-sgTWIST1 cells, the 7 derived clones, and the 4 sgTWIST1 ortho_1 tumors are shown in Supplementary Data 8. Surprisingly, in all the sgTWIST1 clones we detected the same three main indels. Note that three alleles found in the SK-N-Be2c cells, indicating a triploidy of this genomic region as previously described[5].

Neurosphere Assay

NB cells (2x10^4 cells/ml) were cultured plated in duplicate in Neural Crest Stem Cell culture medium (NCSC) [DMEM/F12 (Gibco) supplemented with 1 % penicillin/streptomycin (Gibco), 2 % B27 (Gibco), 20 ng/ml human recombinant bFGF (Peprotech, Rocky Hill, USA), 1% N2 (Gibco), 2-Mercaptoethanol 50µM (AppliChem, Darmstadt, Germany) 15% Chicken Embryo Extract, 20 ng/ml IGF-1 (Peprotech), Retinoic Acid 110 nM], using poly-Hema-coated six wells plates to prevent cell adhesion. After 7 days in culture, pictures of sphere were taken using an Olympus IX53 inverted microscope (Olympus, Shinjuku, Japan) and acquired with the Olympus cellSense imaging software. Spheres were dissociated with StemPro Accutase Cell Dissociation Reagent (Gibco, A11105-01) and the number of cells recovered after the dissociation of spheres was determined using the trypan blue exclusion method.

Proliferation assay

Cell proliferation was assessed using two methods: the MTS/PMS assay and the Trypan Blue assay. For the MTS/PMS assay, $1.2*10^4$ MNA (SK-N-Be2c and LAN-1) and $3*10^4$ no-MNA (NB1-M) cells were seeded in quadruplicate in 96-wells plate in DMEM/FCS. Cell proliferation was assessed using the CellTiter 96® Aqueous Non-radioactive Cell proliferation Assay (Promega, Madison, WI, USA) according to the manufacturer's protocol. For the Trypan Blue assay, SK-N-Be2c cells were seeded in duplicate in 6-wells plate at three different densities: $0.1*10^5$, $0.3*10^5$, $0.5*10^5$ cells/6 well. Cell proliferation was determined by counting manually the live cells after staining with the trypan blue.

Real-Time qPCR

cDNA were prepared from 0.5 or 1 µg of RNA for the validation of human or murine genes, respectively using PrimeScriptTM reagent kit according to the manufacturer's instruction (TAKARA Bio Inc., St.Germain-en-Laye, France). The expression level of selected genes was validated by semi-quantitative real-time PCR in duplicates using primer pairs described in Supplementary Table 3 and the QuantiFast SYBR® green kit (Qiagen, Hilden, Germany). Cycling conditions were: 5 min at 95°C, 40 cycles of 10 sec at 95°C, 30 sec at 60°C, and 1 sec at 72°C with the Rotor Gene 6000 real-time cycler (Corbett, Qiagen). Gene expression levels were determined by normalization to either *HPRT1* (human genes) or *Actb* (murine genes) housekeeping genes using the ΔCt method.

Immunohistochemistry

Histopathological analyses were performed on blocks embedded in paraffin at the Mouse Pathology Facility of Lausanne University (Epalinges, Switzerland) and at the Histology Core Facility of the EPFL (Lausanne, Switzerland). Thin tumor sections (3 µm thick) were de-waxed and rehydrated and then stained with hematoxylin and eosin; Gomori's for reticulin or type III collagen detection, or IHC was performed using primary antibodies (Supplementary Table 4). Staining were imaged using an Olympus BX43 light microscope and then acquired with the Olympus cellSense imaging software or using the EVOS5000 imaging system (Life Technologies); for the Gomori and the VCAN, whole slides were scanned at the 20x magnification using the NanoZoomer S60 Digital slide scanner C13210 (Hamamatsu, Japan). Intrapulmonary metastasis were detected on thin lung section (3 µm thick) by in situ hybridization using the Alu positive probe II (Roche Diagnostics, Cat. No. 05272041001) and the Ventana Discovery ULTRA automate (Roche Diagnostics, Rotkreuz, Switzerland). Whole slides were scanned using the Zeiss Axioscan Z.1 (Zeiss, Oberkochen, Germany). For the quantification of VCAN, F4/80 and Alu positive probe II, the entire surface of either the tumor or three subsequent lung sections separated by a depth of 300 µm, respectively, has been taken into consideration. Quantifications were performed with the QuPath software both

at the magnification of 12.48X. In particular, for the VCAN and F4/80 the QuPath version 0.2.0 has been used according to the following pipeline: select a region of the tumor, analyze, cell analysis, positive cell detection. Options modified from the default parameters for the VCAN quantification: image type: Brightfield (other); detection image: optical density sum; intensity threshold parameters: threshold 1+: 0.3. For the Alu positive II, using the QuPath version 0.2.3 we defined a tight polygonal region around a group of positive cells and then the software automatically calculated its area.

Immunoblotting

NB cells and SK-N-Be2c-derived ortho tumors were lysed in NP40 buffer (50 mM Tris-HCl pH 8.0; 150 mM NaCl; 1% NP-40 and 1x protease inhibitor cocktail (Complete mini, EDTA-free, Roche, Mannheim, Germany). Cell lysates were centrifuged at 15’000 rpm at 4°C for 10 min. Supernatants were recovered and protein concentrations were measured using Bradford method (Bio-Rad Laboratories, Hercules, CA, USA). Equal amount of total protein lysate (40 µg for DE genes validation; 50 µg for TWIST1 and MYCN validation) was loaded onto 4-15% precast polyacrylamide Mini-PROTEAN TGX gel (Bio-Rad Laboratories). Proteins were transferred to PVDF membrane (Immuno Blot,Bio-Rad Laboratories) and blocked with 3% nonfat dry milk in TBS-T 0.1%, incubated overnight at 4˚C with primary antibodies (Supplementary Table 4) and then for one hour with the appropriate secondary antibodies (Supplementary Table 4). Proteins were imaged using either the WesternBright Sirius Kit or the WesternBright Quantum Kit (Advansta Inc., San Jose, CA, USA) and the Fusion FX Spectra multimodal imaging platform (Vilber Lourmat, Marne-la-Vallée, France). Quantification of immunoreactive bands was performed using the ImageJ software according to the following pipeline: analyze, gel, select 1 lane, plot lanes, manually define the area corresponding to each band, wand tool to quantify each selected area.

Supplementary Tables

	Total n (%)	TWIST1				TWIST2			
		Positive tissue n (%)	Mean score	SD	*p* value	Positive tissue n (%)	Mean score	SD	*p* value
**Sample Type**									
Tumor	72	52 (72)	0.84	0.78		30 (42)	1.1	0.87	
Metastasis	25	19 (76)	0.95	0.74		8 (32)	0.31	0.3	
Lymph node	22	18 (82)	1.05	0.72		7 (32)	0.3	0.53	
Liver	3	1 (33)	0.66	0.31		1 (33)	0.75	0.35	
Control tissues	44	0 (0)	0	0		20 (45)	0.58	0.53	
**Age (month)**									
<18 months	27	8 (30)	0.62	0.37	**p*=0.045	16 (59)	0.87	0.53	ns
≥ 18 months	45	33 (73)	0.98	0.53		26 (58)	0.66	0.49	
**INSS stage**									
1	12 (16.7)	4 (33)	0.41	0.19		4 (33)	1.08	0.98	
2	6 (8.3)	3 (50)	0.41	0.24		3 (50)	1.5	0.52	
3	13 (13.8)	10 (77)	1.03	0.48		7 (54)	0.37	0.2	
4	36 (50)	30 (83)	1.1	0.35	**p*=0.04	13 (36)	0.4	0.18	ns
4S	5 (6.9)	2 (40)	0.35	0.12		3 (60)	1.6	1.35	
1, 2	18 (25)	7 (39)	0.42	0.21	***p*<0.01	7 (39)	1.22	0.67	**p*=0.045
3, 4	49 (68)	40 (82)	1.05	0.42		20 (41)	0.4	0.18	
**MYCN amplification**									
MNA	13	11 (85)	1.18	0.35	**p*=0.02	3 (23)	0.17	0.12	***p*<0.01
no-MNA	47	11 (23)	0.6	0.11		22 (47)	0.84	0.38	

Supplementary Table 1: Composition of the TMA and expression of TWIST1 and TWIST2 in NB primary tumors, metastases and control tissues (sympathetic ganglia and adrenal glands), INSS: International Neuroblastoma Staging System, n: number of cases. Mean score: means IHC scores, as established by semi-quantitative analysis of the IHC staining. Student's t-test: *$p<0.05$, **$p<0.01$, $p\geq0.05$ were considered as not significant (ns).

	Gene description	Gene	log2FC	padj
**Myofibroblast markers**	Desmin	Des	1.5436	0.0326
	Mesothelin	Msln	5.0785	7.27E-07
**Sarcomeric thin filament**	Actin, alpha 1, skeletal muscle	Acta1	6.6873	0.0004
	Actinin alpha 2	Actn2	5.0222	0.0035
	Actinin alpha 3	Actn3	5.8876	0.0337
	Myosin binding protein C, slow-type	Mybpc1	6.7193	0.0028
	Nebulin	Neb	4.8947	0.0307
	Nebulin-related anchoring protein	Nrap	7.0611	3.07E-05
	Troponin C2, fast	Tnnc2	6.937	0.0169
	Troponin I, skeletal, fast 2	Tnni2	5.0686	0.0069
	Xin actin-binding repeat containing 2	Xirp2	5.1731	0.0162
**Sarcomeric thick filament**	Cardiomyopathy associated 5	Cmya5	3.8505	0.0337
	Myosin binding protein C, fast-type	Mybpc2	5.9514	0.0087
	Myosin, heavy polypeptide 1, skeletal muscle, adult	Myh1	6.0248	0.0084
	Myosin, heavy polypeptide 4, skeletal muscle, adult	Myh4	6.7812	0.0029
	Myosin, light polypeptide 1	Myl1	5.8567	0.0028
	Myosin light chain, phosphorylatable, fast skeletal muscle	Mylpf	6.2615	0.0075
	Myomesin 2	Myom2	4.3271	0.0096
	Myotilin	Myot	4.3341	0.0273
	Myelin regulatory factor	Myrf	2.7196	0.0035
	Titin-cap	Tcap	5.8251	0.0039
	Titin	Ttn	5.1708	0.0161
**Calcium ion-binding protein**	Parvalbumin	Pvalb	7.9325	0.1618

Supplementary Table 2: Summary of myofibroblast markers and principal muscle structure-specific genes identified in the myofibroblast signature of ortho_1 tumors (labeled in red in **Fig. 8a)** with their log2(FC) and adjusted *p* values.

		Primer sequences (5'-3')	
**Gene**	**Gene description**	**Forward**	**Reverse**
h-*TRIM28*	Tripartite Motif Containing 28	GGA GCC TCT GTG TGA GAC C	CAT CCC GAG ACT TGG CTG G
h-*PDGFRA*	Platelet Derived Growth Factor Receptor Alpha	ACT TCC CAT CCG GCG TTC CT	GGT AAT GAA AGC TGG CAG AG
h-*VCAN*	Versican	GTG ATG CGG GTC TTT ACC GC	CAA GCC TTC TGA GCA GCC TC
h-*ADAMTS19*	A Disintegrin-Like And Metalloprotease With Thrombospondin Type 1 Motif 19	AGC ACC TGA ACA TCT GGC CG	CTA GCC CAG GAC ATT TGC GC
h-*PCOLCE*	Procollagen C-Endopeptidase Enhancer	CCC TGC TGC CTT TTG CCC AG	CCT GAT TCC CCC TTC ACA TC
h-*SYT13*	Synaptotagmin 13	ACT TCA GCG AAG GAG CCA TC	TAA TCA GCA CCA CCA GGA GG
h-*PIRT*	Phosphoinositide Interacting Regulator Of Transient Receptor Potential Channels	CCC TGG GAC ATC GAG GAC AC	GCG CCT AGG ACC AGC AAT AG
h-*HPRT1*	Hypoxanthine Phosphoribosyltransferase 1	TGA CAC TGG CAA AAC AAT GCA	GGT CCT TTT CAC CAG CAA GCT
m-*Pvalb*	Parvalbumin	AAA AGA ACC CGG ATG AGG TG	GAA GCC CTT CAG AAT GGA CC
m-*Neb*	Nebulin	GAG TAC TAC ACA GAG GAG ACA G	GTC CGA TGT CCT TGT CGT TG
m-*Ttn*	Titin	TCA CGT TAG TGG TTC CCC AG	GGG GAT CAT CAA TCT AGC GC
m-*Myh1*	Myosin Heavy Chain 1	GAC CGA AGG CGG AAC TAC TG	GGC TCA TGC AGG TGG GTC AT
m-*Msln*	Mesothelin	GAG ACT GGC AAA GTC AGC G	AAA AGA ACC CGG ATG AGG TG
m-*Marco*	Macrophage Receptor With Collagenous Structure	AGC CAG GAG CAA CTC CGT C	CCC CTT TTG GAC CTG GAG AG
m-*Acta1*	Actin Alpha 1, Skeletal Muscle	TCA CTT CCT ACC CTC GGC AC	GCA CCG GCT TGT TCA CTT GC
m-*Actb*	Actin Beta	GGT GAC AGC ATT GCT TCT G	GAG ACC AAA GCC TTC ATA CAT C

Supplementary Table 3: List of primers used in the study.

Antibody	Species raised	IHC dilution	Agent retrieval	WB dilution	Product Code	Source
VCAN	Rabbit polyclonal	1/200	Citrate, pH=6	-	HPA00476	https://www.sigmaaldrich.com/catalog/product/sigma/hpa004726?lang=fr&region=CH
FAP	Rabbit polyclonal	1/200	Citrate, pH=6	-	ab28244	https://www.abcam.com/fibroblast-activation-protein-alpha-antibody-ab28244.html
F4/80	Rat monoclonal	1/25	Prot K	-	MF48000	F4/80 Antibody (MF48000) (thermofisher.com)
Twist2C1a-ChIP Grade	Mouse monoclonal	-	-	1/100	ab50887	https://www.abcam.com/twist-antibody-twist2c1a-ab50887.html
PCOLCE	Rabbit polyclonal	-	-	1/1000	ab154261	https://www.abcam.com/pcolce-antibody-ab154261.html
KAP1 (TRIM28)	Rabbit polyclonal	-	-	1/1000	ab10484	https://www.abcam.com/kap1-antibody-ab10484.html
ADAMTS19	Rabbit polyclonal	-	-	1/1000	PA5-14351	https://www.thermofisher.com/antibody/product/ADAMTS19-Antibody-Polyclonal/PA5-14351
PIRT	Rabbit polyclonal	-	-	1/300	20990-1-AP	https://www.ptglab.com/products/PIRT-Antibody-20990-1-AP.html
SYT13	Rabbit polyclonal	-	-	1/1000	PA5-32101	https://www.thermofisher.com/antibody/product/SYT13-Antibody-Polyclonal/PA5-32101
MYCN (NCM II 100) ChIP grade	Mouse monoclonal	-	-	1/1000	ab16898	https://www.abcam.com/n-mycmycn-antibody-ncm-ii-100-ab16898.html
ACTB (cl AC-15)	Mouse monoclonal	-	-	1/10000	A-5441	https://www.sigmaaldrich.com/catalog/product/sigma/a5441?lang=fr&region=CH
Peroxidase AffiniPure Goat Anti-Mouse IgG (H+L)	Goat polyclonal	-	-	1/10000	115-035-166	https://www.jacksonimmuno.com/catalog/products/115-035-166
Goat Anti-Rabbit Immunoglobulins/HRP	Goat polyclonal	-	-	1/10000	P0448	Goat Anti-Rabbit Ig/HRP Antibody (affinity isolated) | Agilent

Supplementary Table 4: List of primary and secondary antibodies used in the study.

Supplementary Figures

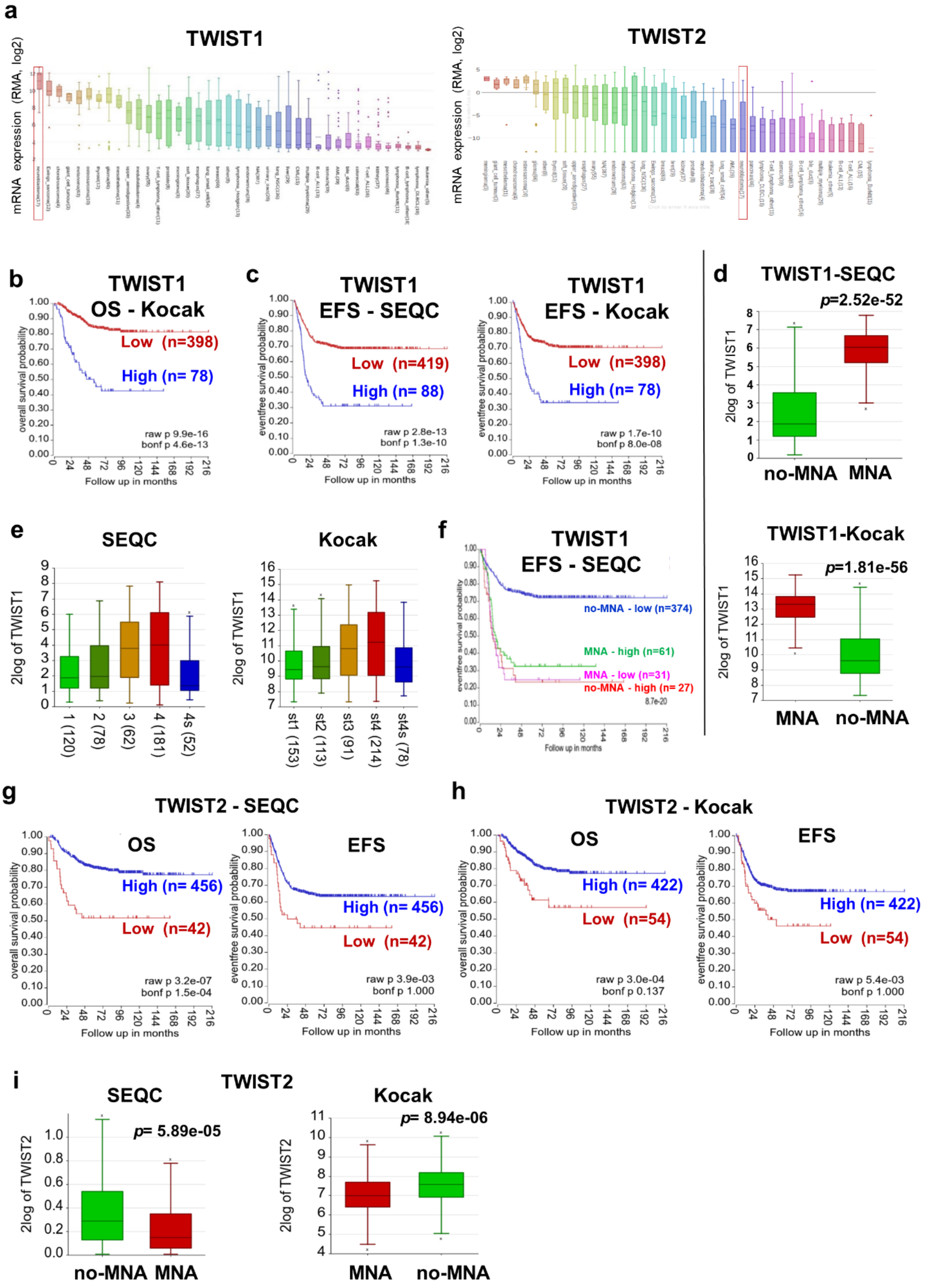

Supplementary Figure 1. ***TWIST1* and *TWITS2* RNA expression in NB cells and tumors**. (**a**) Box plot showing the mRNA expression levels of *TWIST1* (left) and *TWIST2* (right) in a panel of 40 cancer cell lines in the CCLE database. The numbers in the brackets correspond to the numbers of cell lines per tumor types. (**b**) Kaplan-Meier OS curve associated with *TWIST1* expression in the Kocak dataset of primary NB tumors (expression cutoff = 6701.9) (n=476 with survival data). (**c**) EFS associated with *TWIST1* expression in the SEQC (expression cutoff: 44.441) and Kocak (expression cutoff = 6701.9) datasets. (**d** and **e**) Box-and-whisker plots of *TWIST1* expression in MNA and no-MNA tumors (**d**); and in tumors with distinct INSS stages in the indicated datasets (**e**). (**f**) Kaplan-Meier EFS curves showing the stratification of patients of the SEQC dataset according to *MYCN* status and *TWIST1* expression (high or low). (**g** and **h**) OS and EFS according to *TWIST2* expression in the SEQC (**g**, expression cutoff: 1010) and Kocak (**h**, expression cutoff: 86.0) datasets. (**i**) Box-and-whisker plots of *TWIST2* expression in no-MNA and MNA tumors in the SEQC and Kocak datasets.

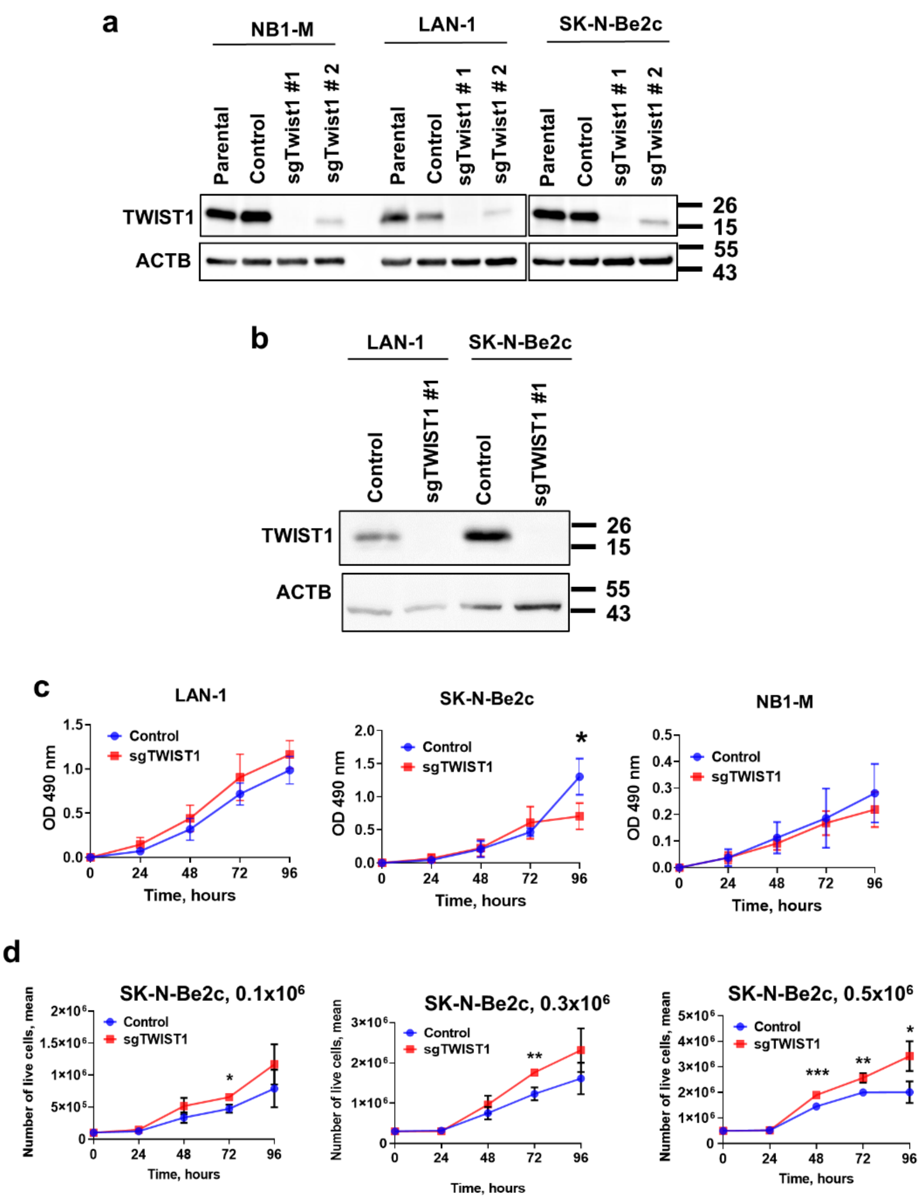

Supplementary Figure 2. Validation of the CRISPR/Cas9-mediated *TWIST1* KO and its impact on NB cell proliferation *in vitro*. (**a**) Immunoblotting for the detection of TWIST1 protein expression and ACTB (as the loading control) in the bulk populations of NB cells before (Parental) and after the lentiviral infection: Control vector, sgTWIST1#1 (sgTWIST1, from now on) and sgTWIST1#2. (**b**) TWIST1 protein expression in the LAN-1 and SK-N-Be2c pool of clones for Control and sgTWST1 (see Methods). (**c**) Cell proliferation of Control and sgTWST1 NB cell lines measured by MTS/PMS assay from 24h to 96h. Mean OD 490nm ± SD of three (SK-N-Be2c) and four (LAN-1 and NB1-M) independent experiments performed in quadruplicates are shown. (**d**) Cell proliferation of SK-N-Be2c-Control and -sgTWIST1 cells measured using trypan blue from 24 to 96 hours. Mean numbers of live cells ± SD of four independent experiments performed in duplicates are shown. Statistical analysis was done in both cases using the Holm-Sidak multiple t-test (α=0.05), without assuming a consistent SD.

Upper panels: *p= 0.0376 in SK-N-Be2c. Lower panels: SK-N_Be2c, $0.1*10^5$: *p= 0.019132 at 72 hours; SK-N_Be2c, $0.3*10^5$: **p= 0.002849 at 72 hours; SK-N_Be2c, $0.5*10^5$: ***p= 0.000818 at 48 hours; **p= 0.005913 at 72 hours; *p= 0.015876 at 96 hours.

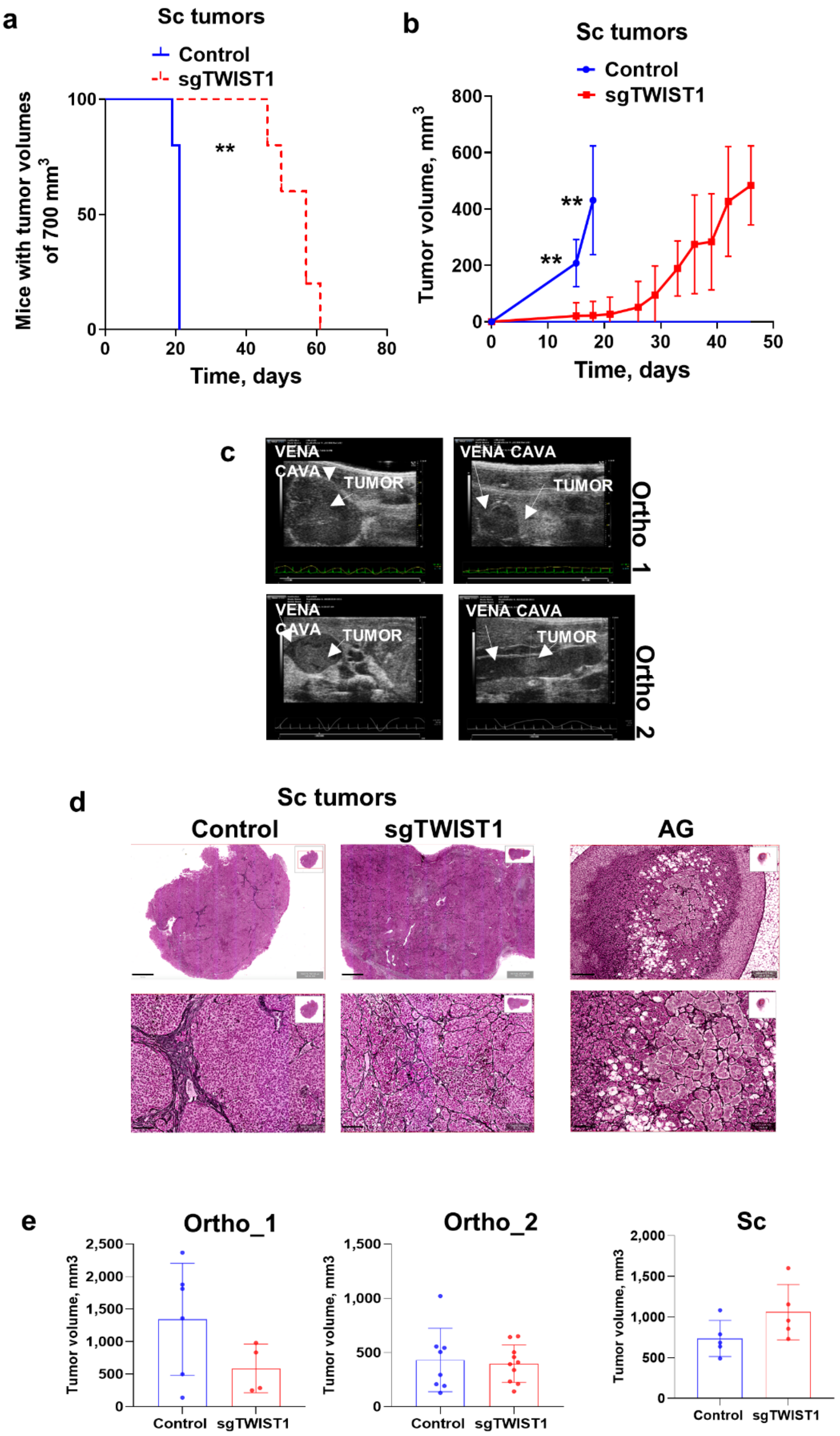

Supplementary Figure 3. *TWIST1* KO diminishes the tumor growth and invasive capacities of SK-N-Be2c cells and affects the Collagen III/reticulin network organization. (**a**) Kaplan-Meier curve indicating the time to reach a tumor volume of ~700 mm^3 in mice

implanted subcutaneously with SK-N-Be2c-Control or -sgTWIST1 cells. Tumor take: 100% (5/5) in both groups. Median survival in the Control vs sgTWIST1 groups: 21 vs 57 days. Gehan-Breslow-Wilcoxon test: ***p=0.0039. (**b**) Tumor growth curve for the sc experiment. Data are plotted as the mean tumor volume ± SD. Mann-Whitney t-test: **p= 0.0079 at both 15 and 18 days. (**c**) Representative ultrasound images of tumor cell intravasation in the vena cava of 2 distinct ortho_1 and ortho_2 Control mice. (**d**) Representative images of Gomori's staining showing the ECM architecture of Control and sgTWIST1 sc tumors (scale bars: top 1mm; bottom 100µm) as compared to the normal AG (scale bars: top 200 µm; bottom 100 µm). (**e**) Graphs illustrating the mean tumor volumes at sacrifice ± SD. Ortho_1: mean Control= 1343 mm^3, n=6; sgTWIST1= 587 mm^3, n=4; Mann-Whitney test: p= 0.26. Ortho_2*:* mean Control= 430 mm^3, n= 8; sgTWIST1= 397 mm^3, n=10; unpaired t-test: p= 0.97. Sc: mean Control: 737 mm^3, n=5; sgTWIST1= 1060 mm^3, n=5; Mann-Whitney: p=0.1.

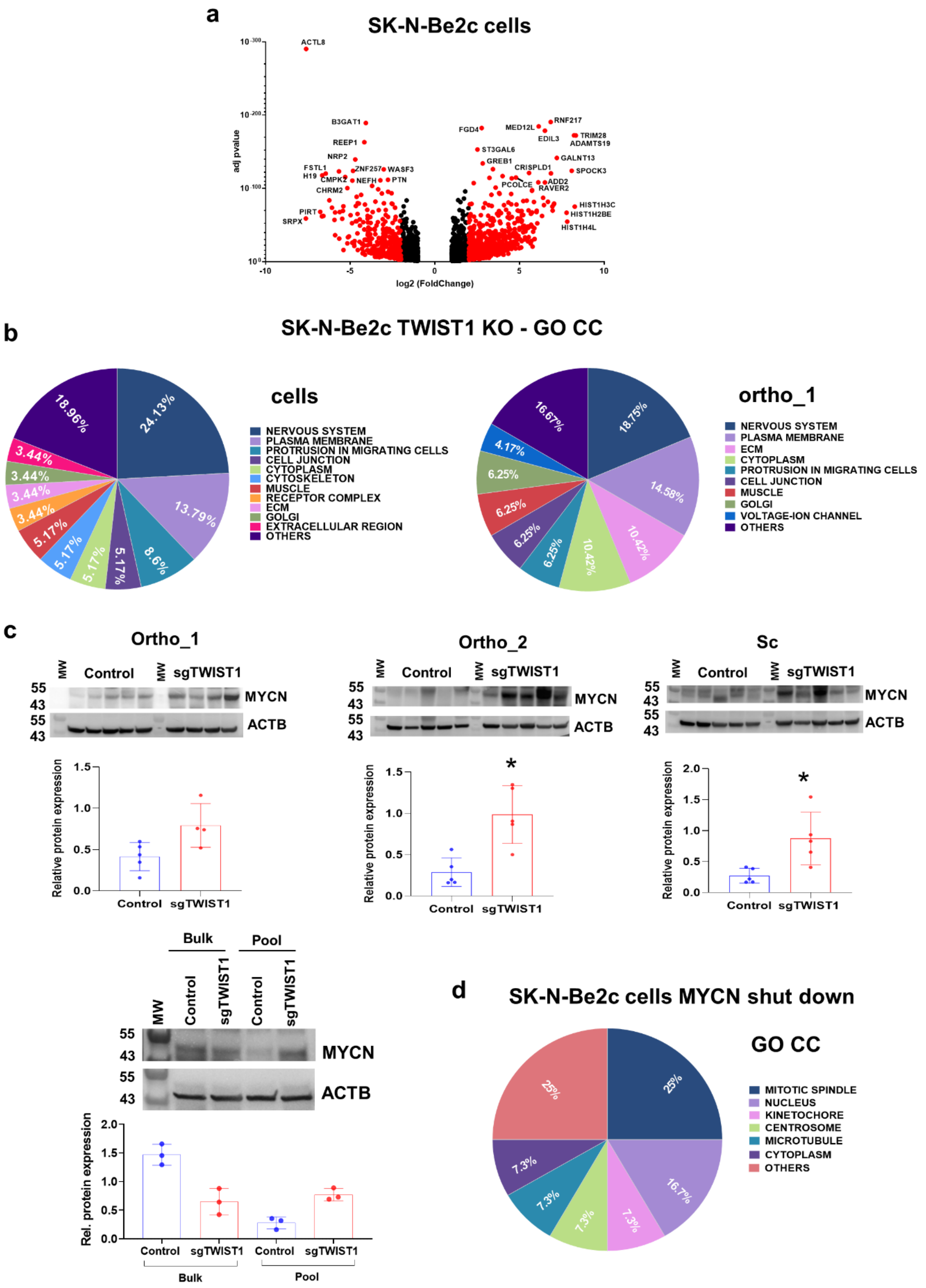

Supplementary Figure 4. Distinct transcriptional programs are affected upon *TWIST1* KO and *MYCN* shut down. (**a**) Volcano plots showing the distribution of the gene expression fold changes and adjusted *p* value for the DE genes in SK-N-Be2c-Control versus SK-N-Be2c–sgTWIST1 cells. Genes with False Discovery Rate (FDR) < 0.05 and absolute value (av) of

log2(FC) ≥ 1 were considered as DE; in red genes with av of log2(FC) ≥2, in black genes with av of log2(FC) ≥ 1 and <2. Positive and negative x-values represent genes either up or down-regulated by TWIST1 respectively. (**b**) Illustration of the cellular components gene sets found enriched by GO analyses (GO CC) in the DE genes following *TWIST1* KO for both SK-N-Be2c cells and ortho_1 tumors. Data are reported as the repartition (in %) of the diverse pathways identified with a FDR < 0.01 (n=58 for cells, n=48 for tumors). (**c**) Immunoblotting for MYCN protein and ACTB as control and densitometric quantification of MYCN expression relative to ACTB in SK-N-Be2c-derived tumors of the 3 *in vivo* experiments and in the bulk transduced SK-N-Be2c cells and the pools of clones. Expressions relative to ACTB were plotted as individual data with mean ± SD. Mann Whitney test. *p= 0.0159 in ortho_2 and sc. Ortho_1: n= 5 Control; n= 4 sgTWIST1; ortho_2 and sc: n= 5 Control; n= 5 sgTWIST1; SK-N-Be2c cells: (n= 3 WB experiments). MW: molecular weight (kDa). (**d**) Illustration of the GO CC gene sets found enriched in the DE genes of SK-N-Be2c cells upon JQ1-mediated MYCN shutdown. RNAseq data of SK-N-Be2c cells treated with JQ1are during 24h or DMSO as control were uploaded (GSE80154, see Methods)[6]. Genes with False Discovery Rate (FDR) < 0.05 and absolute value (av) of log2(FC) ≥ 1 were considered as DE. Data are reported as the repartition (in %) of the diverse pathways identified with a FDR < 0.01 (n=24).

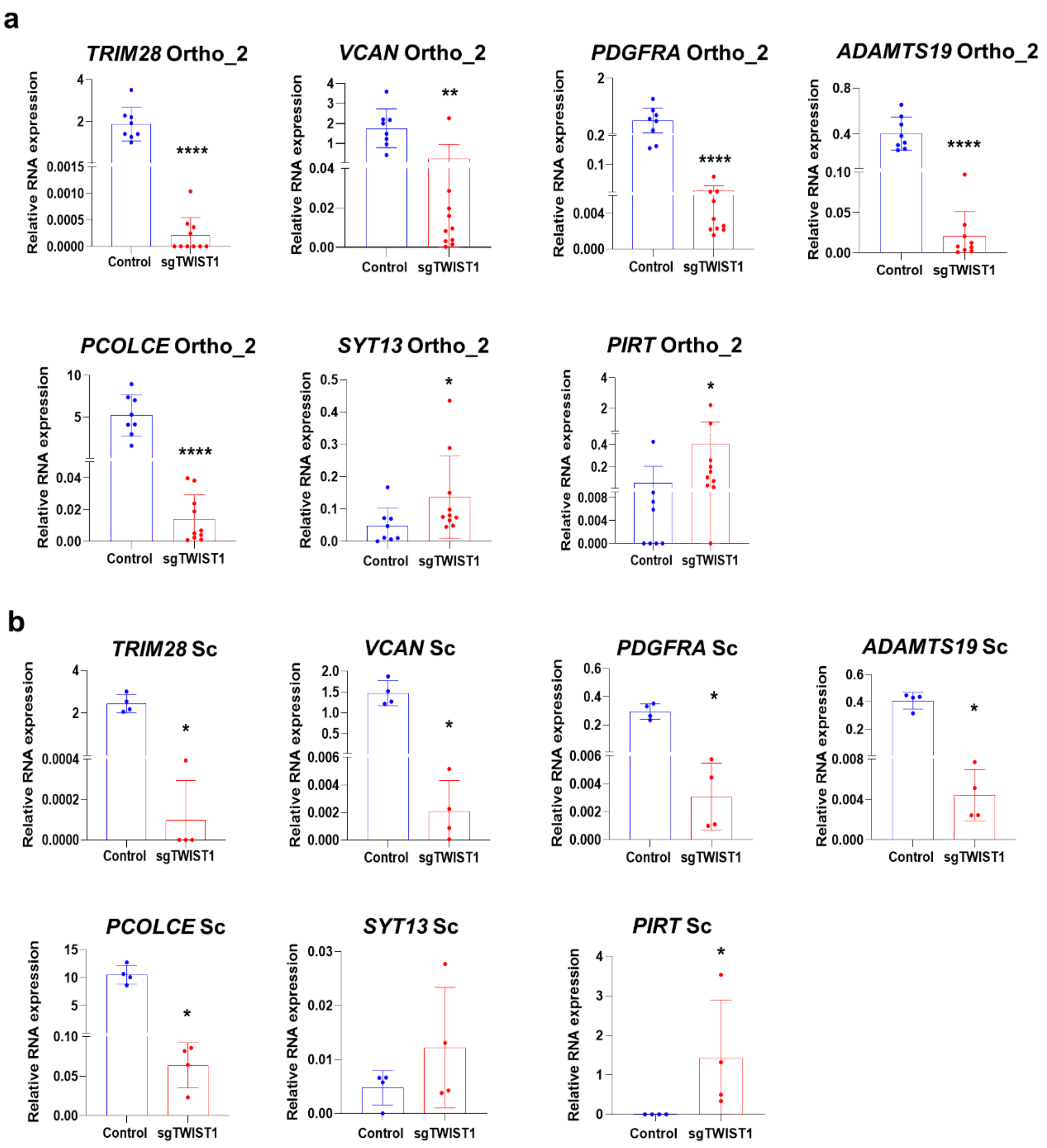

Supplementary Figure 5. Validation of TWIST1-mediated deregulation of selected TWIST1 target genes at the RNA level in SK-N-Be2c-derived tumors. (**a** and **b**) mRNA expression levels of the selected TWIST1 target genes relative to *HPRT1* analyzed by RT-qPCR are plotted as individual values with mean ± SD for the indicated *in vivo* experiments**.** Numbers of tumors analyzed for ortho_2 (**a**): Control n= 8, sgTWIST1 n= 10; and for sc (**b**): Control n= 4, sgTWIST1 n= 4. Statistical analysis was performed using Mann Whitney test for all genes but *PCOLCE in* ortho_2 experiment (unpaired t-test: ****p<0.0001). Ortho_2: *TRIM28*: ****p=0.0001 2; *VCAN*: **p=0.0021; *PDGFRA* and *ADAMTS19*: ****p=0.0001; *PIRT*: *p=0.0146; *SYT13*: *p=0.0266; sc: *TRIM28*: *p=0.; *VCAN*, *PDGFRA*, *ADAMTS19* and *PIRT*: *p=0.0286; *SYT13*: *p=0.0266.

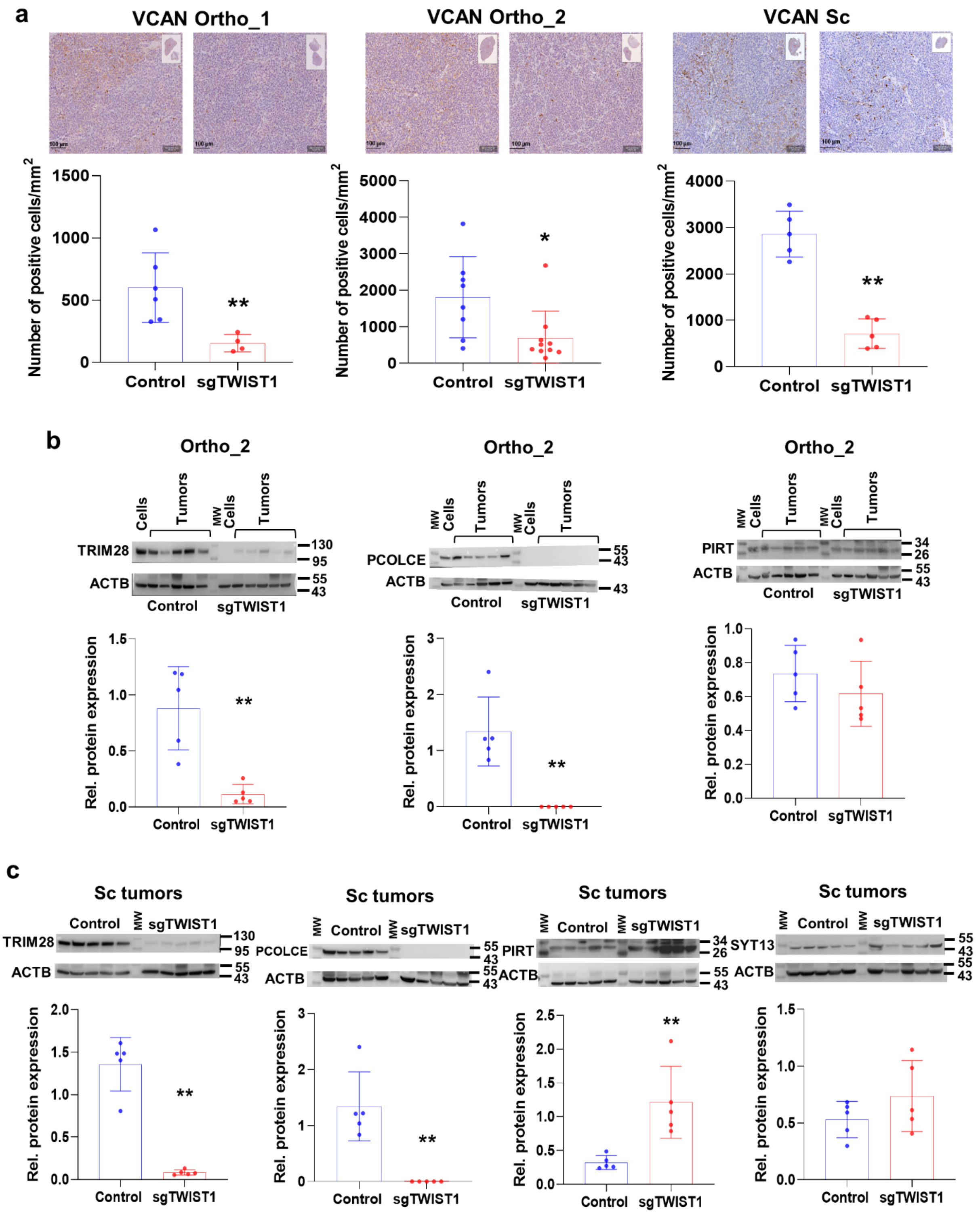

Supplementary Figure 6. Analysis of the protein expression levels of selected TWIST1 target genes in SK-N-Be2c-derived tumors. (**a**) Upper panels: representative images of IHC for VCAN in the indicated tumors (scale bar =100 μm); lower panels: quantification of VCAN staining (Qpath software on total area of each section). Mann-Whitney: ortho_1:***p*=0.0095, n=6 Control and n=4 sgTWIST1; ortho_2: **p*= 0.0155, n=8 Control and n=10 sgTWIST1; sc: ***p*=0.0079, n=5 Control and n=5 sgTWIST1. (**b** and **c**) Relative protein expression as

determined by immunoblotting for the selected genes in the ortho_2 (**b**) and the sc (**c**) tumors. Representative images of immunoblotting for TRIM28, PCOLCE, PIRT and SYT13 (ACTB as the control) and densitometric quantifications of immunoreactive band densities. Expressions relative to ACTB were plotted as individual data with mean ± SD. Mann Whitney test: **p=0.0079 for all comparisons. Ortho_2 and sc: n=5 Control and n=5 sgTWIST1. MW: molecular weight (kDa).

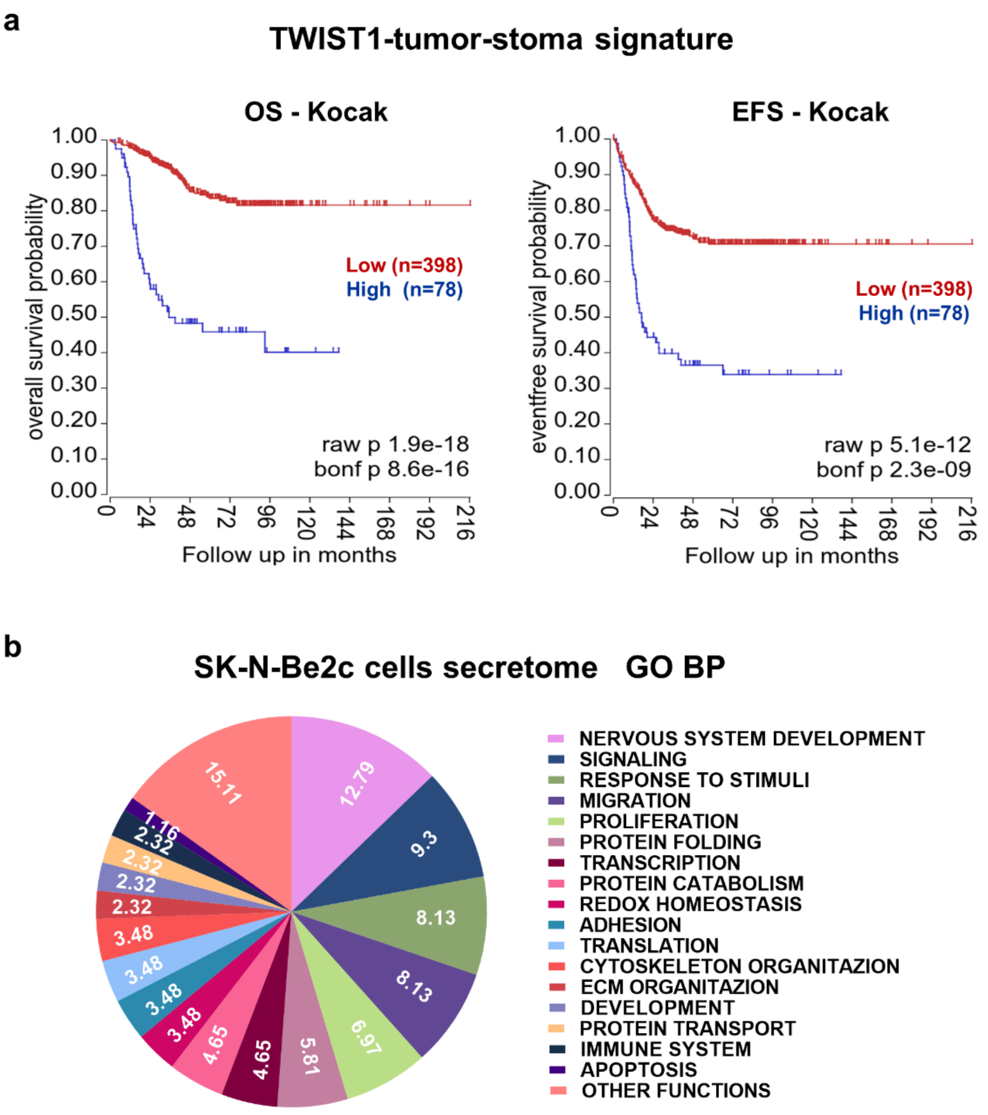

Supplementary Figure 7. Correlation between the TWIST1-tumor-stroma signature in the Kocak NB dataset and the outcome of patients. (**a**) Kaplan-Meier plots showing the correlation between a high level of the paracrine signature expression and a poor OS and EFS of NB patients in the Kocak dataset. Expression cutoff for both curves: 0.14. (**b**) Illustration of biological processes (BP) found enriched by gene ontology analysis for the DE proteins in SK-N-Be2c cell secretome. Data are reported as the repartition (in %) of the diverse BP identified with a FDR < 0.01 (n=50).

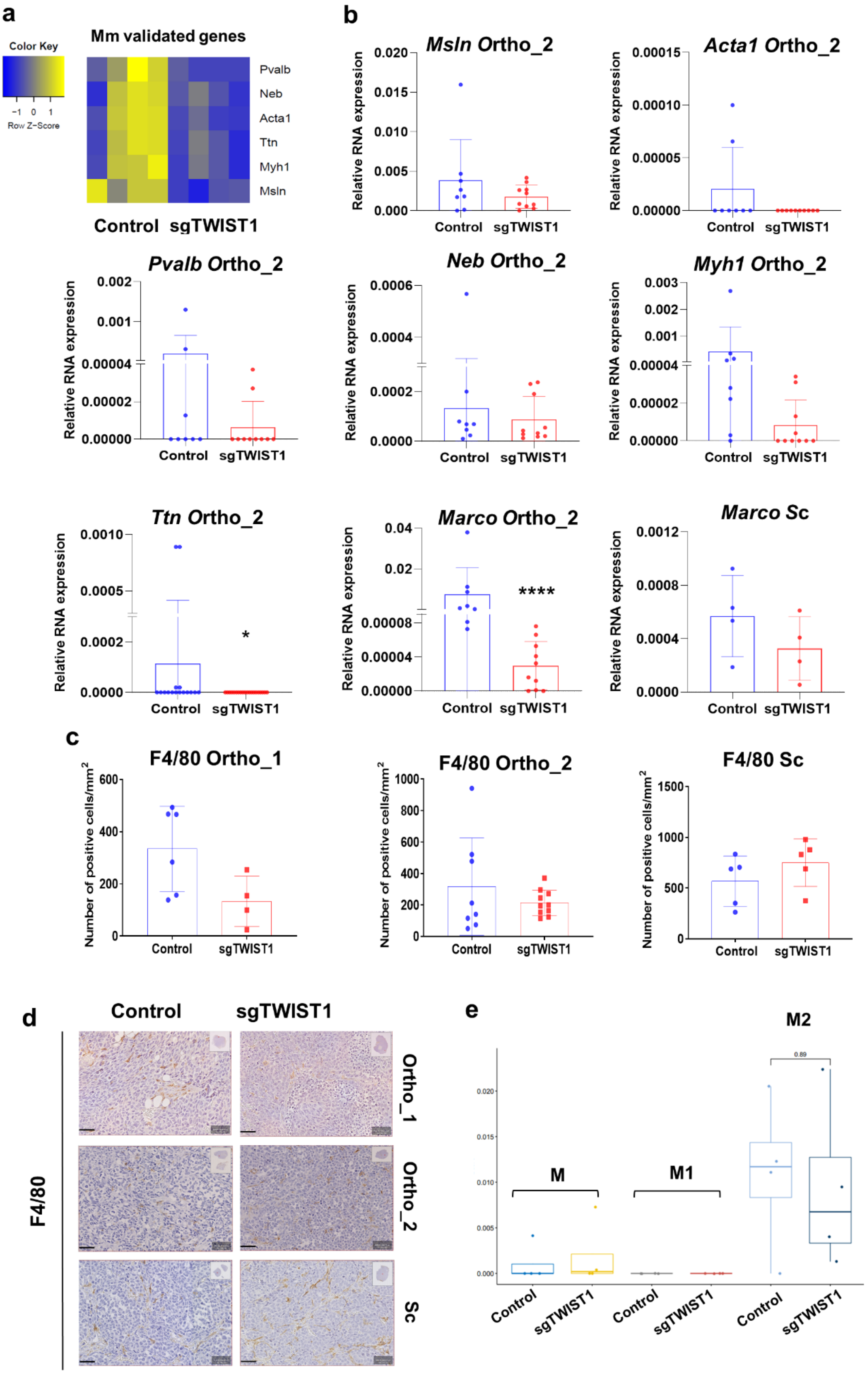
a
Color Key
Row Z-Score
Mm validated genes
Pvalb
Neb
Acta1
Ttn
Myh1
Msln
Control sgTWIST1
b
Msln Ortho_2
Acta1 Ortho_2
Pvalb Ortho_2
Neb Ortho_2
Myh1 Ortho_2
Ttn Ortho_2
Marco Ortho_2
Marco Sc
Relative RNA expression
Control
sgTWIST1
c
F4/80 Ortho_1
F4/80 Ortho_2
F4/80 Sc
Number of positive cells/mm2
d
F4/80
Ortho_1
Ortho_2
Sc
e
M
M1
M2
0.89

Supplementary Figure 8. **Validation of selected genes of the myofibroblast signature in the ortho_2 and sc tumors and quantifications of the macrophage populations.** (**a**) Heatmap showing the RNA expression levels (z-score) of the Myofibroblasts signature selected genes as determined by RNAseq analysis in ortho_1 tumors. (**b**) mRNA expression levels for the selected myofibroblast genes and *Marco* relative to *Actb* as determined by RT-qPCR. Data are plotted as individual values with mean ± SD. Mann Whitney test: Ortho_2: *Ttn*: *p= 0.0309; *Marco*: ****p<0.0001. Ortho_2 tumors: Control n=8; sgTWIST1 n=10; sc tumors: Control n=4, sgTWIST1 n=4. (**c**) Quantification of F4/80 staining (Qpath software on total area of tumor sections). Ortho_1: n=6 Control and n=4 sgTWIST1; ortho_2: n=8 Control and n=10 sgTWIST1; sc: n=5 Control and n=5 sgTWIST1. (**d**) Representative images of IHC for F4/80 in the indicated tumors (20X, scale bar =50 µm). (**e**) Macrophages M, M1 and M2 signature scores calculated using xCell on the ortho_1 RNAseq data for all genes belonging to the murine stroma (n=4 samples).

a

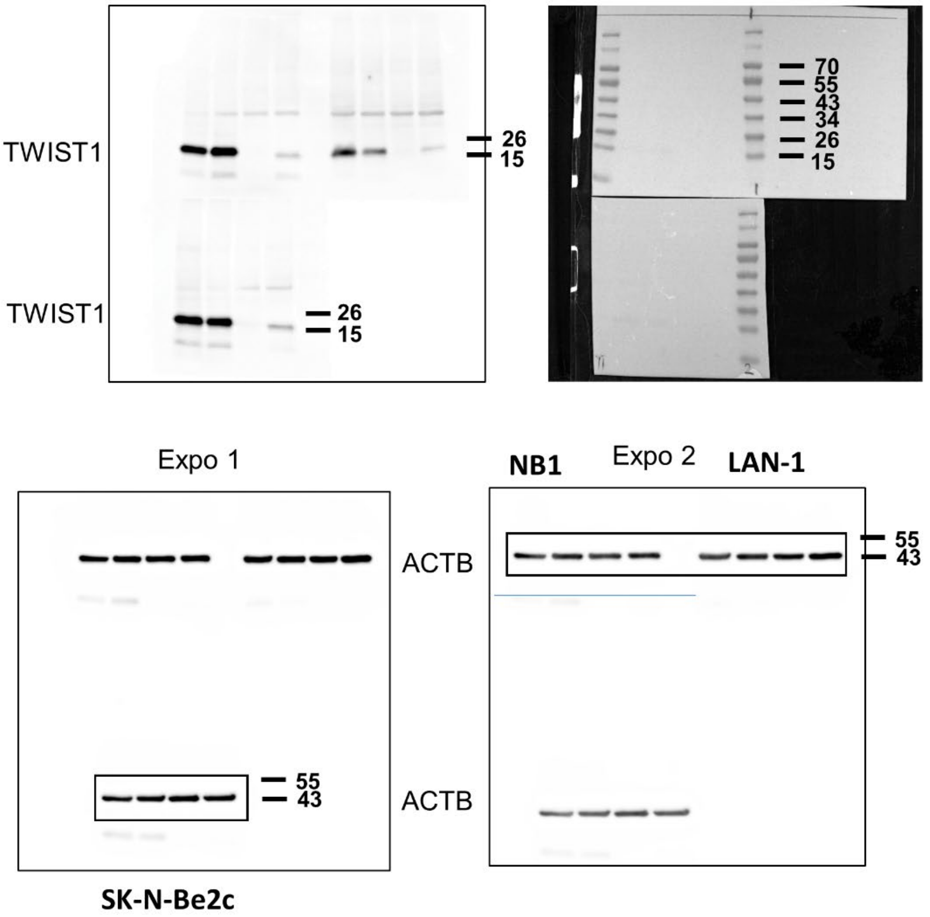

b

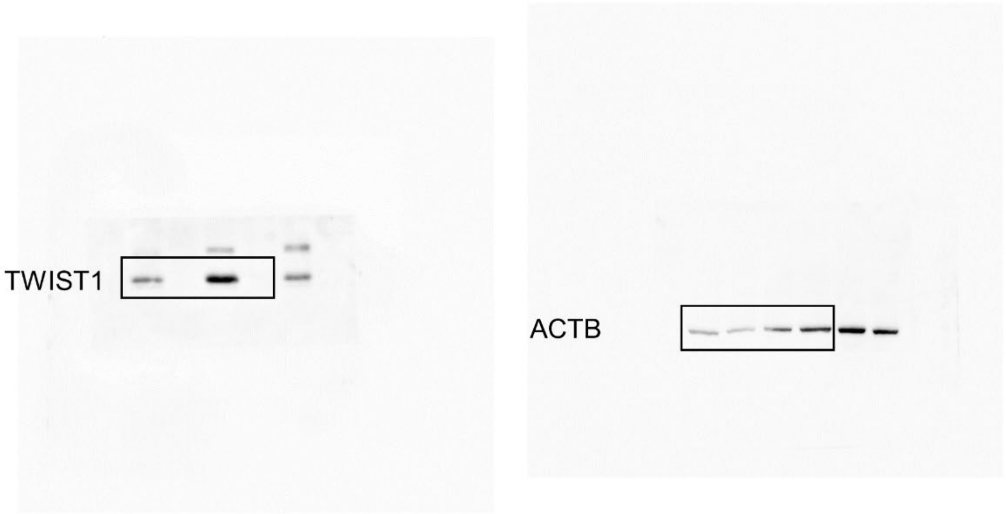

Supplementary Figure 9. Uncropped blot corresponding to Supplementary Fig. 2a-b.

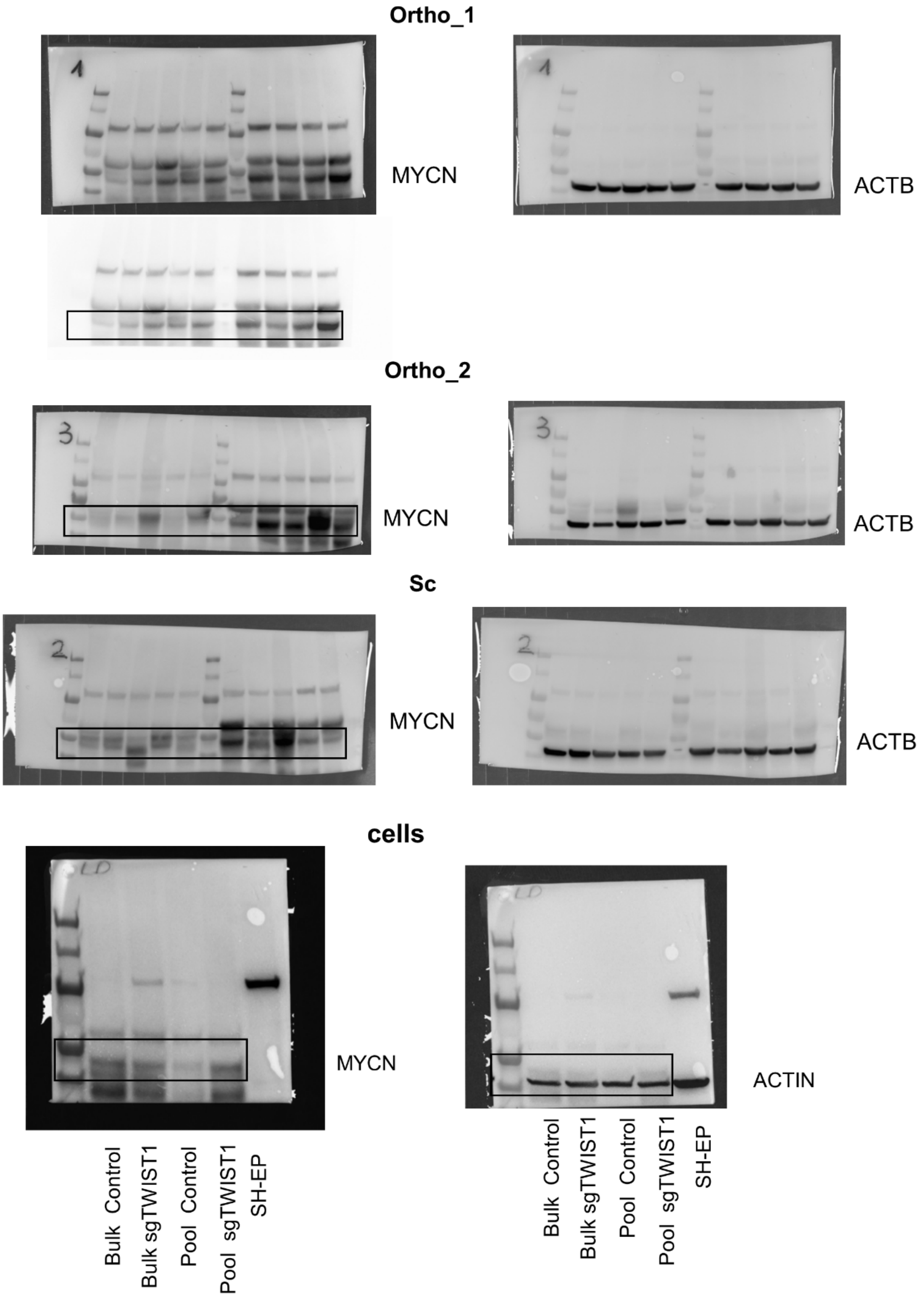

Supplementary Figure 10. Uncropped blot corresponding to Supplementary Fig. 4c.

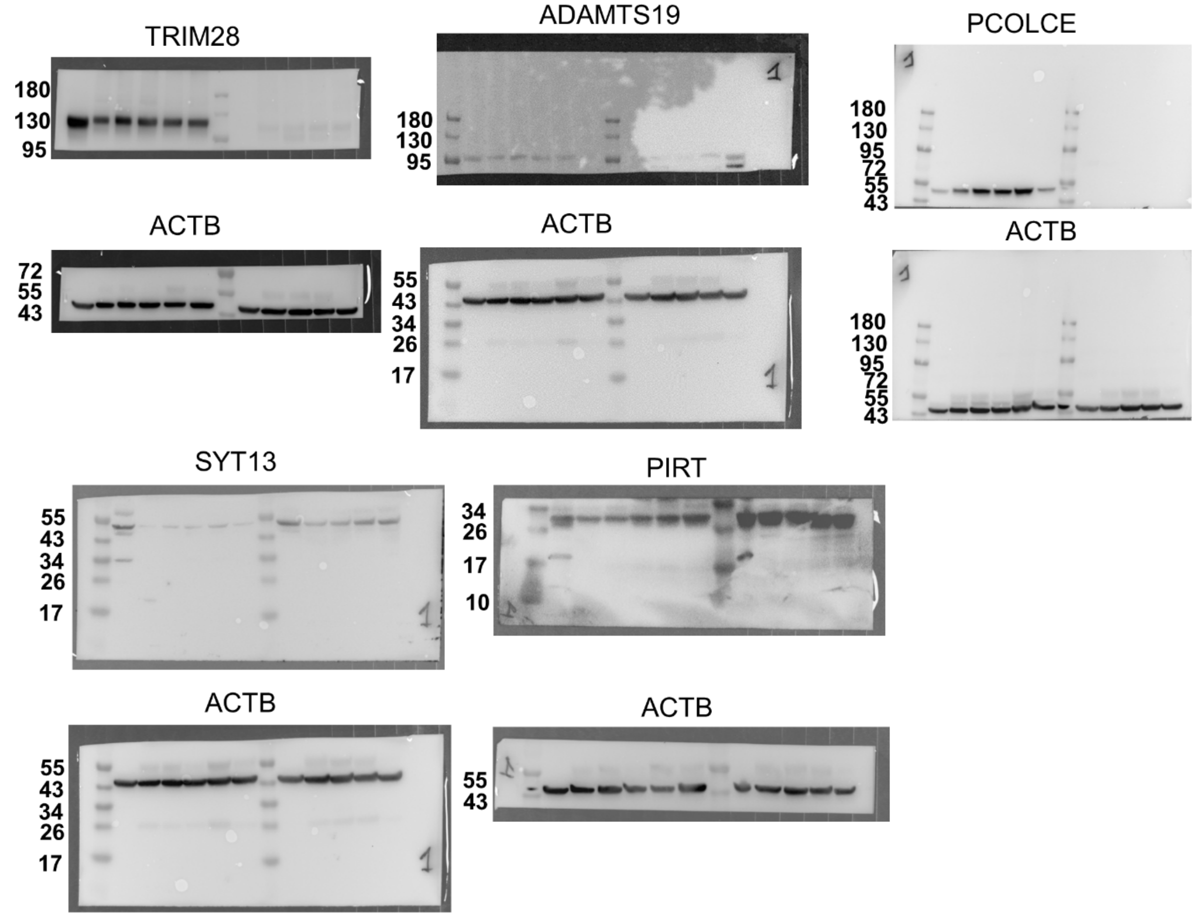

Supplementary Figure 11. Uncropped blot corresponding to Fig. 6c.

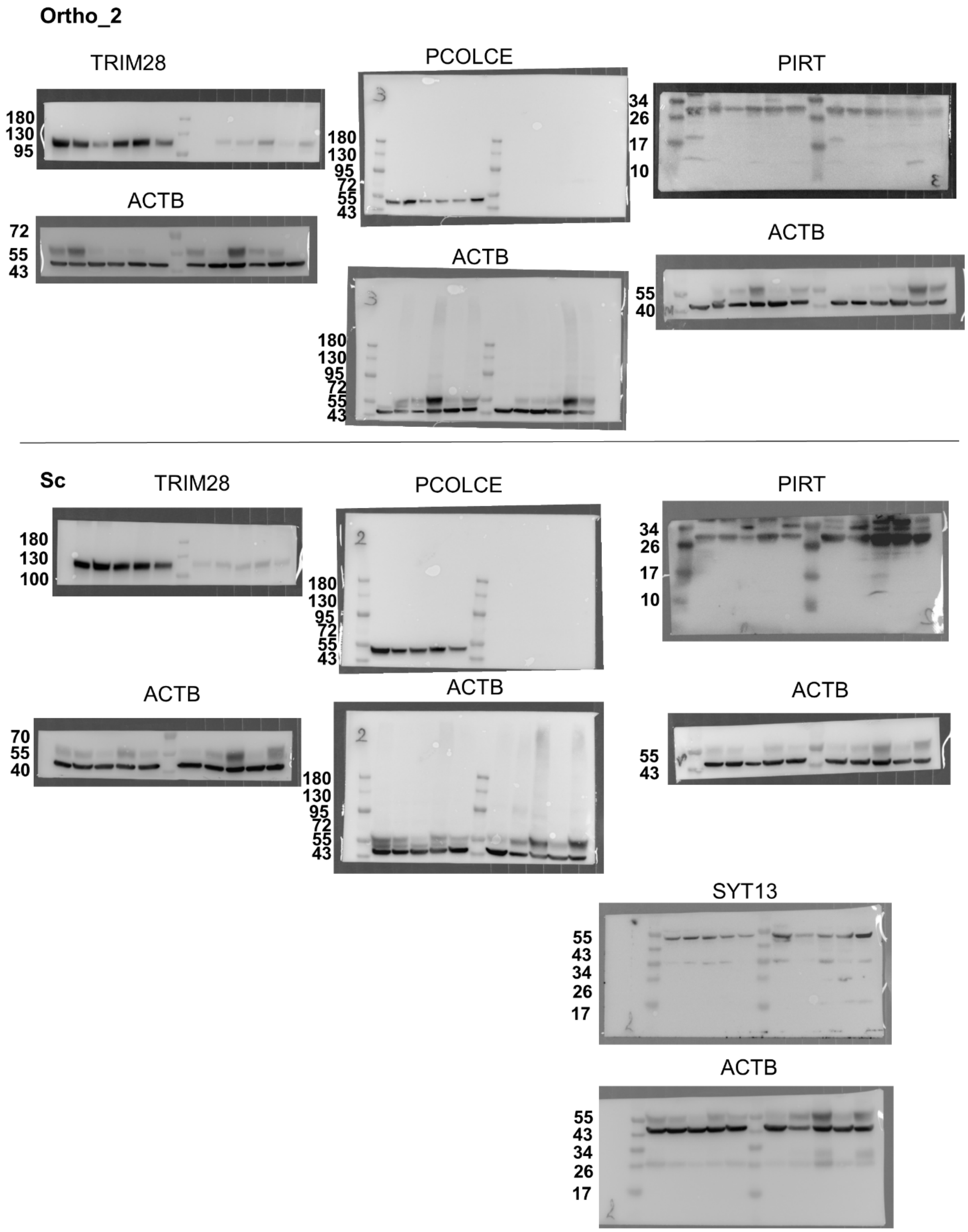

Supplementary Figure 12. Uncropped blot corresponding to Supplementary Fig. 6b-c.

Supplementary References

1 Shalem, O. *et al.* Genome-scale CRISPR-Cas9 knockout screening in human cells. *Science (New York, N.Y.)* **343**, 84-87, doi:10.1126/science.1247005 (2014).

2 Sanjana, N. E., Shalem, O. & Zhang, F. Improved vectors and genome-wide libraries for CRISPR screening. *Nature methods* **11**, 783-784, doi:10.1038/nmeth.3047 (2014).

3 Goldschneider, D. *et al.* Expression of C-terminal deleted p53 isoforms in neuroblastoma. *Nucleic acids research* **34**, 5603-5612, doi:10.1093/nar/gkl619 (2006).

4 Valsesia-Wittmann, S. *et al.* Oncogenic cooperation between H-Twist and N-Myc overrides failsafe programs in cancer cells. *Cancer cell* **6**, 625-630, doi:10.1016/j.ccr.2004.09.033 (2004).

5 Carr, J. *et al.* High-resolution analysis of allelic imbalance in neuroblastoma cell lines by single nucleotide polymorphism arrays. *Cancer genetics and cytogenetics* **172**, 127-138, doi:10.1016/j.cancergencyto.2006.08.012 (2007).

6 Zeid, R. *et al.* Enhancer invasion shapes MYCN-dependent transcriptional amplification in neuroblastoma. *Nature genetics* **50**, 515-523, doi:10.1038/s41588-018-0044-9 (2018).
