# Supplementary material for: TWIST1 expression is associated with high-risk neuroblastoma and promotes primary and metastatic tumor growth
Source: Commun Biol. 2022 Jan 12;5:42. doi: 10.1038/s42003-021-02958-6 (PMC8755726; doi:10.1038/s42003-021-02958-6)
Supplement: Supplementary file 2 — Description of Additional Supplementary Files [file 42003_2021_2958_MOESM2_ESM.pdf]

Description of Additional Supplementary Files

File name: Supplementary Data 1
Description: List of all human protein coding genes detected by RNAseq, with DE genes in SK-N-Be2c-Control and -sgTWIST1 cells, ortho_1 tumors, and in common between cells and ortho_1 tumors.

File name: Supplementary Data 2
Description: List of pathways identified upon TWIST1 KO by GO analysis in SKN-Be2c cells and ortho_1 tumors.

File name: Supplementary Data 3
Description: List of raw counts, DE genes deregulated by MYCN in SK-N-Be2c cells after MYCN shutdown with JQ1 for 24h (GSE80154) and pathways identified by GO analysis.

File name: Supplementary Data 4
Description: Gene lists for the identification of the TWIST1-signature.

File name: Supplementary Data 5
Description: List of the 77 genes of the TWIST1-tumor-stroma signature.

File name: Supplementary Data 6
Description: List of all proteins detected in the secretome of SK-N-Be2c-Control and -sgTWIST1 cells, the DE proteins and the deregulated pathways.

File name: Supplementary Data 7
Description: List of all murine protein coding genes detected by RNAseq in SK-N-Be2cControl and -sgTWIST1 ortho_1 tumors, the 89 DE genes and the pathways identified by GO analysis.

File name: Supplementary Data 8
Description: Illustration of the insertions/deletions generated in the TWIST1 gene.
