# Supplementary material for: TWIST1 expression is associated with high-risk neuroblastoma and promotes primary and metastatic tumor growth
Source: Commun Biol. 2022 Jan 12;5:42. doi: 10.1038/s42003-021-02958-6 (PMC8755726; doi:10.1038/s42003-021-02958-6)
Supplement: Supplementary file 11 — Reporting Summary [file 42003_2021_2958_MOESM11_ESM.pdf]

nature research

Corresponding author(s): Mühlethaler-Mottet Annick

Last updated by author(s): November 19, 2021

Reporting Summary

Nature Research wishes to improve the reproducibility of the work that we publish. This form provides structure for consistency and transparency in reporting. For further information on Nature Research policies, see our Editorial Policies and the Editorial Policy Checklist.

Statistics

For all statistical analyses, confirm that the following items are present in the figure legend, table legend, main text, or Methods section.

n/a	Confirmed	
☐	☒	The exact sample size (*n*) for each experimental group/condition, given as a discrete number and unit of measurement
☐	☒	A statement on whether measurements were taken from distinct samples or whether the same sample was measured repeatedly
☐	☒	The statistical test(s) used AND whether they are one- or two-sided<br>*Only common tests should be described solely by name; describe more complex techniques in the Methods section.*
☒	☐	A description of all covariates tested
☐	☒	A description of any assumptions or corrections, such as tests of normality and adjustment for multiple comparisons
☐	☒	A full description of the statistical parameters including central tendency (e.g. means) or other basic estimates (e.g. regression coefficient) AND variation (e.g. standard deviation) or associated estimates of uncertainty (e.g. confidence intervals)
☐	☒	For null hypothesis testing, the test statistic (e.g. *F*, *t*, *r*) with confidence intervals, effect sizes, degrees of freedom and *P* value noted<br>*Give P values as exact values whenever suitable.*
☒	☐	For Bayesian analysis, information on the choice of priors and Markov chain Monte Carlo settings
☐	☒	For hierarchical and complex designs, identification of the appropriate level for tests and full reporting of outcomes
☒	☐	Estimates of effect sizes (e.g. Cohen's *d*, Pearson's *r*), indicating how they were calculated

Our web collection on statistics for biologists contains articles on many of the points above.

Software and code

Policy information about availability of computer code

Data collection	Detailed in Methods section

Data analysis

1. In silico transcriptomic data were analyzed on the R2: Genomics Analysis and Visualization Platform (https://hgserver1.amc.nl/cgi-bin/r2/main.cgi).

2. Sequence alignments (RNA-Seq) to the reference genomes (Hg19, Mm10) was performed using STAR version 2.5.2. Per-gene quantification using rsem version 1.3.0. All RNAseq per-gene data quality checks and analysis were done in R. Principal Component Analysis were done using the normalized log2 (rpkm) values. Differential analyses were performed using the raw data counts in DESeq2 package version 1.26.0. Functional gene ontology analysis was performed by applying a hypergeometric test on selected genes lists against gene sets from GO (Molecular Function, Biological Process and Cellular Component), KEGG, REACTOME, or BIOCARTA pathways.

3. Tandem MS data were processed by the MaxQuant software (version 1.6.3.4) incorporating the Andromeda search engine and MaxQuant data were further processed with Perseus software for the filtering, log2-transformation, normalization of values and the statistical analyses. T-test corrected for FDR with the Benjamini-Hochberg method and a threshold q-value at 0.02 was used for the statistical analysis.

4. Proteins were imaged using the Fusion FX Spectra multimodal imaging. Quantification of immunoreactive bands was performed using the ImageJ 1.52 software

5. Visualization and analyses of the IHC scans was performed using the QuPath v0.2.0-m9 and v0.2.3 imaging software.

6. All statistical analyses were performed using GraphPadPrism 8.3.0.

7. PCR amplicons were checked for indels using CRISPResso (https://crispresso.pinellolab.partners.org)

For manuscripts utilizing custom algorithms or software that are central to the research but not yet described in published literature, software must be made available to editors and reviewers. We strongly encourage code deposition in a community repository (e.g. GitHub). See the Nature Research guidelines for submitting code & software for further information.

Data

Policy information about availability of data

All manuscripts must include a data availability statement. This statement should provide the following information, where applicable:

- Accession codes, unique identifiers, or web links for publicly available datasets
- A list of figures that have associated raw data
- A description of any restrictions on data availability

All data analyzed and/or generated during this study are included in the article and its Supplementary Information files. The RNAseq, proteomic and image datasets generated in this study can be accessed at: the GEO public repository, using the accession number GSE160765; and the ProteomeXchange public repository using the accession number PXD024200; and the images datasets at the Zenodo repository with the doi: 10.5281/zenodo.565852465 and doi: 10.5281/zenodo.565834566, respectively. The RNAseq data of SK-N-Be2c JC1 samples were obtained from GEO GSE80153. All other data that support the findings of this study are available from the corrisponding author upon reasonable request.

Field-specific reporting

Please select the one below that is the best fit for your research. If you are not sure, read the appropriate sections before making your selection.

☒ Life sciences ☐ Behavioural & social sciences ☐ Ecological, evolutionary & environmental sciences

For a reference copy of the document with all sections, see nature.com/documents/nr-reporting-summary-flat.pdf

Life sciences study design

All studies must disclose on these points even when the disclosure is negative.

Sample size

In vivo experiments included data from n=5-12 mice for each group depending on the experiments, as indicated in the Figure Legends. Data are reported as mean with standard deviation in the graphs and Figure Legends.

Data exclusions

Three mice of the Control group of the ortho_2 experiments died before the first echography for tumor measument could take place. Thus, these mice were excluded for the survival curve analysis. No other samples have been excluded.

Replication

All in vitro tests were performed with n>=3 independent experiments with 2 to 5 technical replicates for each experiment as specified in the Figure/Supplementary Figure legends for each assay. All attempts at replication were succesful. RNAseq analyses were performed in biological triplicates for the cells and quadruplicates for tumors; secretome analysis was done in triplicates. The number of animals for RNA and protein validations is described in the corresponding Figure Legends.

Randomization

Mice were cohoused for at least two weeks prior to the start of the experiment and were randomly allocated to experimental groups before injection.

Blinding

Two independent investigators were blinded for the TMA analysis. All mice surgeries were blinded for the cell type injection. IHC staining were blinded for the tissue grouping. In all other cases, the investigators were not blinded as they were responsible for both experimental design and data collection. We followed standard laboratory procedures of randomization. Each experiment was designed with proper controls, and samples for comparison were collected and analyzed under the same conditions and at the same time whenever possible.

Reporting for specific materials, systems and methods

We require information from authors about some types of materials, experimental systems and methods used in many studies. Here, indicate whether each material, system or method listed is relevant to your study. If you are not sure if a list item applies to your research, read the appropriate section before selecting a response.

Materials & experimental systems

n/a	Involved in the study
☐	☒ Antibodies
☐	☒ Eukaryotic cell lines
☒	☐ Palaeontology and archaeology
☐	☒ Animals and other organisms
☐	☒ Human research participants
☒	☐ Clinical data
☒	☐ Dual use research of concern

Methods

n/a	Involved in the study
☒	☐ ChIP-seq
☒	☐ Flow cytometry
☒	☐ MRI-based neuroimaging

Antibodies

Antibodies used

All antibody with catalog numbers are provided in the Supplementary Table 4

Validation

-Abcam antibodies validation steps : https://www.abcam.com/primary-antibodies/how-we-validate-our-antibodies.
-Proteintech antibodies validation steps: https://www.lubio.ch/supplier/proteintech/#:~:text=Proteintech%20are%20currently%20developing%20their,still%20an%20%20important%20target%20today.
-Sigma antibodies validation steps: https://www.sigmaaldrich.com/technical-documents/articles/biology/antibody-enhanced-validation.html.
-Thermo Fisher antibodies validation steps: https://www.thermofisher.com/ch/en/home/life-science/antibodies/invitrogen-antibody-validation.html.
Information about host species, reactivity, and applications are freely available from manufacturer's websites.
1. VCAN: https://www.sigmaaldrich.com/catalog/product/sigma/hpa004726?lang=fr&region=CH;
2. FAP: https://www.abcam.com/fibroblast-activation-protein-alpha-antibody-ab28244.html;
3. F4/80: https://www.thermofisher.com/antibody/product/F4-80-Antibody-clone-BM8-Monoclonal/MF48000
4. TWITS1: https://www.abcam.com/twist-antibody-twist2c1a-ab50887.html;
5. PCOLCE: https://www.abcam.com/pcolce-antibody-ab154261.html;
6.KAP1 (TRIM28): https://www.abcam.com/kap1-antibody-ab10484.html;
7.ADAMTS19: https://www.thermofisher.com/antibody/product/ADAMTS19-Antibody-Polyclonal/PA5-14351;
8.PIRT: https://www.ptglab.com/products/PIRT-Antibody-20990-1-AP.html;
9.SYT13: https://www.thermofisher.com/antibody/product/SYT13-Antibody-Polyclonal/PA5-32101;
10.MYCN: https://www.abcam.com/n-mycmycn-antibody-ncm-ii-100-ab16898.html;
11. ACTB: https://www.sigmaaldrich.com/catalog/product/sigma/a5441?lang=fr&region=CH;
12. Peroxidase AffiniPure Goat Anti-Mouse IgG (H+L): https://www.jacksonimmuno.com/catalog/products/115-035-166;
13. Goat Anti-Rabbit Immunoglobulins/HRP: https://www.agilent.com/en/product/immunohistochemistry/antibodies-controls/secondary-antibodies/goat-anti-rabbit-immunoglobulins-hrp-(affinity-isolated)-153244.

Eukaryotic cell lines

Policy information about cell lines

Cell line source(s)

All cell lines and their sources are provided in the methods section "Cell lines

Authentication

Authentication of SK-N-Be2c and LAN1 cell lines was performed by microsatellite short tandem repeat analysis (Microsynth, Switzerland)

Mycoplasma contamination

All cell lines were tested negative for mycoplasma contamination

Commonly misidentified lines
(See ICLAC register)

No misidentified cell lines were used in this study

Animals and other organisms

Policy information about studies involving animals; ARRIVE guidelines recommended for reporting animal research

Laboratory animals

Athymic Swiss nude mice (Crl:NU(Ico)-Foxn1nu (female: 6-7 weeks old for the orthotopic injections; 18 weeks old for the subcutaneous injection) were purchased from Charles River Laboratory, France and housed at the Animal Facility of the FBM-UNIL under 12-h light dark cycles, controlled temperature (~23 °C) and 40~50% humidity with free access to food and water.

Wild animals

The study did not involve wild animals.

Field-collected samples	The study did not involve samples collected from the fields.
Ethics oversight	Guidelines for animal care of the Swiss Animal Protection Ordinance and the Animal Experimentation Ordinance of the Swiss Federal Veterinary Office (FVO). Animal experimentation protocols were approved by the Swiss FVO.

Note that full information on the approval of the study protocol must also be provided in the manuscript.

Human research participants

Policy information about studies involving human research participants

Population characteristics	Children patients diagnosed with NB between July 1988 and November 2001, treated and followed at the Bicêtre hospital (Le Kremlin-Bicêtre) and the Gustave Roussy Institute (Villejuif), enrolled in SIOPEN studies.
Recruitment	The TMA samples were choosen to reflect the proportion of MYCN amplified samples in NB.
Ethics oversight	This study was approved by the local ethics committee for the Canton de Vaud

Note that full information on the approval of the study protocol must also be provided in the manuscript.
